# Agro‐ecological zone and farm diversity are factors associated with haemoglobin and anaemia among rural school‐aged children and adolescents in Ghana

**DOI:** 10.1111/mcn.12643

**Published:** 2018-07-25

**Authors:** Fusta Azupogo, Elisabetta Aurino, Aulo Gelli, Kwabena M. Bosompem, Irene Ayi, Saskia J. M. Osendarp, Inge D. Brouwer, Gloria Folson

**Affiliations:** ^1^ Division of Human Nutrition Wageningen University Wageningen The Netherlands; ^2^ Department of Family and Consumer Sciences, Faculty of Agriculture University for Development Studies Tamale Ghana; ^3^ Centre for Health Economics and Policy Innovation, Department of Management Imperial College Business School, Imperial College London London UK; ^4^ International Food Policy Research Institute (IFPRI) Washington District of Columbia; ^5^ Noguchi Memorial Institute for Medical Research (NMIMR), College of Health Sciences University of Ghana Legon Greater Accra Region Ghana

**Keywords:** adolescents, agro‐ecological zone, anaemia, Ghana, Haemoglobin (Hb), school‐aged children

## Abstract

Understanding contextual risk factors for haemoglobin (Hb) status and anaemia of rural school‐aged children (SAC) and adolescents is critical in developing appropriate interventions to prevent anaemia. We analysed secondary data from the baseline of an impact evaluation of the Ghana School Feeding Programme to determine the severity of anaemia and contextual factors associated with anaemia and Hb status among rural SAC (6–9 years; *n* = 323) and adolescents (10–17 years; *n* = 319) in Ghana. We used regression models with variable selection based on backward elimination in our analyses. The mean Hb was 113.8 ± 13.1 g/L, and the overall prevalence of anaemia was 52.3%, being 55.1% and 49.5% among SAC and adolescents, respectively. We identified child's age (β = 2.21, *P* < 0.001); farm diversity score (β = 0.59, *P* = 0.036); and agro‐ecological zone (*P* trend <0.001) as the main predictors of Hb of SAC. Household asset index (*P* trend = 0.042) and agro‐ecological zone (*P* trend <0.001) were predictors of Hb in adolescents. Agro‐ecological zone and age were predictors of anaemia, but the effect of age was only significant for girls and not boys (prevalence odds ratio [POR] = 1.35, 95% CI [1.04, 1.76] vs. POR = 1.14, 95% CI [0.88, 1.46]). SAC in households with maize stock were less likely to be anaemic (POR = 0.55, 95% CI [0.32, 0.97]). Household dietary diversity score (β = 0.59, *P* = 0.033) was associated with Hb status for the full sample only. Anaemia is a severe public health problem among SAC and adolescents in rural Ghana irrespective of sex. Farm diversity score, availability of maize stock in the household, household asset index, and agro‐ecological zone were the main predictors of Hb and anaemia among the rural SAC and adolescents.

**Key messages**

- Anaemia is a severe public health problem among rural SAC and adolescents in Ghana irrespective of sex.
- Farm diversity, availability of maize stock in the household, HAI, and agro-ecological zone were the main contextual factors associated with Hb and anaemia.
- A unit increase in age was associated with *reduced odds* of anaemia for SAC and *higher odds* of anaemia for, particularly adolescent girls.
- Given the severity of anaemia in other agro-ecological zones compared with the forest zone, iron and folate supplementation may be prioritized in these zones for both SAC and adolescents alongside both boys and girls.

## 1 INTRODUCTION

Anaemia is a critical public health condition globally, particularly in low‐ and middle‐income countries where its prevalence tends to be 3 to 4 times higher than in advanced economies (McLean, Egli, De Benoist, Wojdyla, & Cogswell, 2007; WHO, 2008). Besides pregnant women, children (both preschool and school age) are the most affected group with iron‐deficiency anaemia because of the rapid growth and the general cognitive development (Kassebaum et al., 2015). Studies have shown that adolescents are also at an increased risk of developing anaemia due to increased iron demand during puberty, menstrual losses, limited dietary iron intake, and poor dietary habits (Chaparro & Lutter, 2008; WHO/FAO, 2004). Adolescent girls are particularly at high risk of developing iron deficiency and/or anaemia, especially among those who experience heavy blood losses during menstruation and corresponding decreases in ferritin levels (Black et al., 2008; Chaparro & Lutter, 2008).

It is estimated that about half of school‐aged children (SAC; 6–9 years) and pregnant women and close to one third of nonpregnant women of reproductive age suffer from anaemia globally (McLean et al., 2007). In 21 countries assessed by UNICEF, more than one third of adolescent girls were anaemic (UNICEF, 2012). In Ghana, about 73.6% and 44% of rural young children (<5 years) and women are anaemic, respectively (Ghana Statistical Service/Ghana Health Service, 2015). About two thirds of Ghanaian adolescent girls and SAC are reportedly anaemic (Abizari, Azupogo, & Brouwer, 2017; UNICEF, 2012), suggesting anaemia is a severe public health problem in Ghana requiring urgent attention and context‐appropriate policies.

The consequences of iron deficiency and anaemia have been well documented; these include impairment of physical and cognitive development of infants and young children (Batra & Sood, 2005; Chang, Zeng, Brouwer, Kok, & Yan, 2013; Jáuregui‐Lobera, 2014), higher risk of morbidity and mortality for young children and pregnant women (Sanghvi, Ross, & Heymann, 2007; Semba & Bloem, 2008), and increased risk of low birth weight even with moderate preconception anaemia on the part of women in fertile age (Bhutta et al., 2013). The long‐term effect of anaemia is reduced cognition in the early years, which is associated with lower productivity later in life (Haas & Brownlie, 2001; WHO, 2008). In fact, the negative consequences of iron deficiency or anaemia on cognitive performance may not be limited only to infants and young children but may continue into adolescence. In a randomized controlled trial with iron supplementation, Bruner, Joffe, Duggan, Casella, and Brandt (1996) showed that iron‐deficient American adolescent high school girls receiving a 1,300‐mg daily dose of ferrous sulphate for 8 weeks performed better on a test of verbal learning and memory than girls with a similar iron status receiving a placebo.

The primary cause of anaemia is assumed to be iron deficiency; nevertheless, this condition is seldom present in isolation (WHO, 2008). This is because anaemia is only partly caused by dietary insufficiency of nutrients and it coexists with infectious diseases such as malaria and parasitic infections, haemoglobinopathies as well as socio‐economic and environmental factors such as geographical location, the source of drinking water and sewage system; these determinants are also often context specific (Darnton‐Hill & Mkparu, 2015; Syed et al., 2016; Tesfaye, Yemane, Adisu, Asres, & Gedefaw, 2015; WHO, 2008).

Given the context specificity of the root causes of anaemia, understanding contextual risk factors is critical to guide the implementation of interventions to prevent or reduce anaemia. Nevertheless, there is generally a dearth of data on the major predictors of anaemia among SAC and adolescents as existing studies focus primarily on preschool children (<5 years) or women of reproductive age. Hence, this study aims to fill this critical gap by secondary analyses of data from an impact evaluation to determine the prevalence, severity, and contextual factors associated with anaemia and haemoglobin (Hb) status among rural Ghanaian SAC and adolescents and provide evidence for policy formulation and programme planning.

## 2 MATERIALS AND METHODS

### 2.1 Study design

We used baseline data from the impact evaluation of the Ghana School Feeding Programme conducted in December 2013. Ethical approval for the impact evaluation was obtained from the Institutional Review Board of the University of Ghana Noguchi Memorial Institute for Medical Research (NMIMR) and Imperial College London Research Ethics Committee. Written and verbal informed consent was obtained from all parents/guardians of the children before the interviews. Details of the impact evaluation study design and of the data collected have been reported elsewhere (Gelli et al., 2015).

### 2.2 Study population and sample size

The study population in the impact evaluation was selected through cluster random sampling from the Greater Accra, Ashanti, Brong Ahafo, Central, Eastern, Northern, and Upper East Regions of Ghana. In the impact evaluation, a random subsample of 717 children aged 4–17 years had measurements of Hb taken. Seventy‐five (75) children not meeting the inclusion criteria (age ≥ 6 years and residing in a rural community) were excluded, which led to the final sample of 642 children aged 6–17 years for the analyses.

### 2.3 Measurements

#### 2.3.1 Hb and anaemia

Hb was measured with HemoCue Hb 201^+^ using blood samples from a finger prick test by a trained phlebotomist. Anaemia was defined with the WHO criteria (WHO, 2011): Hb < 115 g/L for 6‐ to 11‐year‐old children; Hb < 120 g/L for 12‐ to 14‐year‐old children; Hb < 130 g/L for males ≥15 years; and Hb < 120 g/L for nonpregnant females ≥15 years. We also categorized anaemia as severe (Hb < 80 g/L), moderate (Hb 80–109 g/L), and mild (Hb 110–119 g/L) using the WHO criteria (WHO, 2011).

#### 2.3.2 Helminths infestation

Urine and stool samples were collected separately from a subsample of the children (*N* = 311) in order to screen them for ascaris, hookworm, trichuriasis, and schistosomiasis. Analyses were carried out by microscopy and/or RDT at the General Laboratory of the Parasitology Department of NMIMR. We defined helminths infestation as the presence of any of the above parasites, and a dummy variable was created.

#### 2.3.3 Household dietary diversity

A modified household dietary diversity score (HDDS; Swindale & Bilinsky, 2006) was calculated for each household using data on consumption of 68 food items grouped into 12 food groups, over the previous 7 days. For each food group, a score of 1 was given if a household consumed at least one food item from the food group in the past week, else 0. The food group scores were summed into the modified HDDS, ranging from 0 to 12. Food groups included cereals, roots and tubers, vegetables, fruits, meat, eggs, fish and seafood, pulses and nuts, milk and milk products, oils and fats, sugar, and condiments. Furthermore, a household food variety score was constructed similar to the food variety score (Savy, Martin‐Prevel, Sawadogo, Kameli, & Delpeuch, 2005), which consisted of the sum of all the unique food items consumed by a household from the 7‐day recall. Similar to the HDDS, the household food variety score was a continuous score ranging 0–68. Lastly, we created a score for household animal food consumption (HAFC) consisting of a sum of all the unique animal foods consumed by the household from the 7‐day food recall; the HAFC ranged 0–9. These scores did not consider minimum quantities of intakes.

#### 2.3.4 Farm diversity

A composite crop and livestock count (“farm diversity”) captured the number of the different crops cultivated and of the different animals reared by the household in the last farming season. This count indicator has previously been used to assess farm diversity and biodiversity in relation to nutritional outcomes (Jones, Shrinivas, & Kerr, 2014; Sibhatu, Krishna, & Qaim, 2015).

#### 2.3.5 Other covariates

The household questionnaire included modules on household demographics, education, economic activities, and farm income. The data included information on household durables and agriculture assets. We constructed household and agricultural assets indices through principal component analysis (Filmer & Pritchett, 2001) and then divided them into terciles. These indices captured household wealth in relation to the ownership of durable and agricultural assets. Furthermore, we included in our statistical models a continuous variable for the proportion of food consumed by the household from own production in the past month.

We included in the initial models the following continuous variables: the birth order of the child; the number of days the child bought food from school; the age of the household head; the household's dependency ratio; the number of months the household consumed food from own production; and parental years of schooling. Whereas paternal occupation was coded and analysed as farmer or other (which included off‐farm casual paid job and unemployed), maternal occupation was coded and analysed as farmer, trader, and other (employed, off‐farm casual job, and apprentice) based on the data available. Additionally, we analysed the following as dichotomous variables: availability of maize stock in the house, land ownership, receipt of any form of remittance in the past year, sex of household head, and child sickness in the 7 days preceding the survey. A categorical variable comprised four agro‐ecological zones in Ghana (Oppong‐Anane, 2006; Owusu & Waylen, 2009), namely, Northern Savannah (Northern and Upper East Regions), Coastal Savannah (Central Region), transition zone (Brong Ahafo Region), and forest zone (Ashanti and Eastern Regions). We re‐coded ethnicity as Akan, Gurma, Mole‐Dagbani, and other (including Guan, Grusi, Ga‐Dangbe, Ewe, and other ethnic groups).

Children were categorized as school age (6–9 years) and adolescents (10–17 years) based on their age (UNICEF, 2011). Lastly, school grade was categorized as lower primary and upper primary/junior high school.

### 2.4 Statistical analysis

We used SAS 9.3 (SAS Institute Inc., Cary NC.) in all our statistical analyses and a two‐tailed *P* value ≤0.05 was considered statistically significant. Population characteristics were presented as means ± *SD* for continuous variables and percentages for categorical variables. We used one‐way analysis of variance to assess the difference in means between groups for continuous outcome variables whereas Pearson's chi‐square test was employed for categorical variables. Based on the literature, we identified an a priori set of potential predictors of Hb and anaemia. These included child sex and age, helminths infestation, child health status (including malaria), dietary intake, household size, educational status of household head and of the mother, the occupation of the household head and of the mother, household wealth, and ethnicity (Bharati, Shome, Chakrabarty, Bharati, & Pal, 2009; Mesfin, Berhane, & Worku, 2015; Salama & Labib, 2016; Tesfaye et al., 2015). The assumption of normality for linear regression was assessed by visual inspection (Q–Q plots, histograms, and boxplots), and normality violations were corrected by a natural log transformation (proportion of food consumed from own production in the past month) before analysis. The scale of continuous covariates in the logistic regression was assessed with smoothed scatter plots.

We included variables with *P* values ≤0.25 from bivariate analyses along with those thought to be important a priori (sex, parental education, and occupation) in backward stepwise multiple regression models using the GLMSELECT PROCEDURE (Mcgahan & Chong, 2009) and PROC LOGISTIC PROCEDURE (SAS Institute Inc, 2008) in SAS to, respectively, determine the regression coefficients (β) and the prevalence odds ratios (PORs) of the significant predictors of Hb status and anaemia in the sample. The criteria for variable selection in the GLMSELECT PROCEDURE was based on the significance level and the Predicted Residual Sum of Squares Statistic whereas the significance level and log‐likelihood ratio test were used for the logistic regression. We entered interaction terms in order to explore potential non‐linearities, but none of these interactions was statistically significant.

The analysis was also stratified for child's age category (SAC and adolescents). Lastly, as a robustness check, we repeated the analysis with multilevel regression with a random intercept and clustering at the agro‐ecological zone using the PROC MIXED PROCEDURE in SAS (Littell, Milliken, Stroup, Wolfinger, & Schabenberger, 2006).

## 3 RESULTS

### 3.1 Sample characteristics

Table 1 presents the sample characteristics. About 50.3% of the children were of school age and the majority attended lower primary school. About half were females. Children's ethnicity included Akan (38.9%), Gurma (25.9%), Mole‐Dagbani (17.6%), and other (17.6%). About 11.5% of the children reported being sick 7 days preceding the survey, and none was found to have helminths infestation. Generally, the dependency ratio was high. Furthermore, the mean paternal and maternal years of schooling were 5.6 ± 6.5 and 3.1 ± 5.6 years, respectively. About 72% of the children belonged to male‐headed households. Farming was the main occupation of both parents, but SAC children had more farmer fathers compared with adolescents. A greater proportion of SAC were in lower primary school compared with adolescents. In addition, households of SAC had more land and a higher farm diversity compared with adolescent households. Also, the proportion of food consumed from own production was lower for households of SAC, but their households consumed food from their own production for a longer duration compared with adolescent households. Lastly, more SAC than adolescents were from the Northern Savannah agro‐ecological zone.

**Table 1:** Household and child level characteristics of the rural schoolchildren stratified by age category

Characteristics	Overall *n* = 642	School‐aged children (6–9 years), *n* = 323	Adolescents (10–17 years), *n* = 319
Child characteristics			
Sex (female), %	48.6	48.3	48.9
Age, y	9.5 ± 2.2	7.7 ± 1.1	11.3 ± 1.3
Haemoglobin concentration (g/L),	113.8 ± 13.1	111.4 ± 12.8	116.2 ± 12.9
Any anaemia, %	52.3	55.1	49.5
Category of anaemia, %			
Mild	15.7	13.3	18.2
Moderate	36.0	40.6	31.4
Severe	0.6	1.2	0
Birth order of child (median)	5	5	5
Number of days child bought food from school	3.6 ± 2.2	3.4 ± 2.3	3.7 ± 2.1
Helminths infestation present (n = 311), %	0	0	0
Child sick in the past 7 days (*n* = 589), %	11.5	12.5	10.5
Child's school grade, %			
Lower primary	83.6	97.8	69.3
Upper primary and junior High school	16.4	2.2	30.7
Ethnicity of child, %			
Akan	39.1	37.5	40.8
Gurma	25.9	28.2	23.5
Mole‐Dagbani	17.6	17.0	18.2
Other^a^	17.5	17.3	17.6
Household demographic and socio‐economic characteristics			
Dependency ratio	0.6 ± 0.2	0.6 ± 0.2	0.6 ± 0.2
Sex of household head, %			
Male	72.0	72.1	71.8
Female	28.0	27.9	28.2
Age of household head	45.9 ± 13.1	45.6 ± 12.7	46.2 ± 13.4
Paternal years of schooling	5.6 ± 6.5	5.4 ± 6.5	5.7 ± 6.6
Maternal years of schooling	3.1 ± 5.6	2.8 ± 5.4	3.5 ± 5.8
Household receipt of remittance in the past 1 year, %	29.6	26.4	32.9
Occupation of father (*n* = 429), %			
Famer	69.2	77.1	60.7
Other^b^	30.8	22.9	39.3
Occupation of mother (*n* = 596), %			
Farmer	45.1	47.3	43.0
Trader	37.6	35.5	39.7
Other^c^	17.3	17.2	17.3
Household asset index, %			
Lower	34.2	35.9	32.9
Middle	32.9	32.2	33.5
Upper	32.7	31.9	33.5
Household food availability and diet diversity			
HDDS	9.3 ± 1.9	9.3 ± 1.9	9.3 ± 1.9
HFVS	19.2 ± 6.1	19 ± 5.8	19.5 ± 6.3
HAFC	1.9 ± 1.2	1.8 ± 1.0	1.9 ± 1.3
Proportion of food consumed in the past month from own production^d^	1.7 ± 1.7	1.5 ± 1.6	1.9 ± 1.8
Number of months household consumed food from own production	6.9 ± 4.2	7.5 ± 3.9	6.3 ± 4.4
Maize stock available in household, %	23.6	24.4	22.3
Farm diversity	3.5 ± 2.6	3.7 ± 2.6	3.2 ± 2.6
Agriculture asset index, %			
Lower	47.5	45.8	49.2
Middle	34.4	34.4	34.5
Upper	18.1	19.8	16.3
Household owns land, %	78.3	81.7	74.9
Geographical location			
Ecological zone, %			
Northern Savannah	29.6	34.4	24.8
Coastal Savannah	9.0	7.1	11.0
Transitional	22.9	21.1	24.8
Forest	38.5	37.5	39.5

*Note*. Unless specified, values are mean ± *SD*; *N*: sample size; HDDS: household dietary diversity score; HFVS: household food variety score; HAFC: household animal foods consumption.

a Other includes Ga‐Dangbe, Guan, Grusi, Mande, Ewe, and other tribes originating from outside Ghana.

b Other includes off‐farm wage employment, business, and unemployed.

c Other includes off‐farm wage employment, apprentice, and unemployed.

d Natural log‐transformed variable of proportion of food consumed in the past month from own production.

The mean Hb concentration of the children in the study was 113.8 ± 13.1 g/L and was significantly lower for SAC compared with adolescents (SAC: 111.4 ± 12.8 g/L; adolescents: 116.2 ± 12.9 g/L, *P* < 0.001; Table 1). The overall prevalence of anaemia was 52.3% and did not differ between SAC and adolescents (SAC: 55.1%; adolescents: 49.5%, *P* = 0.157). However, more SAC were moderately anaemic compared with adolescents and although about 1.2% of SAC were severely anaemic, none of the adolescents was severely anaemic (Table 1).

### 3.2 Results of the bivariate analyses

We found a significant variation (*P* < 0.001) in the mean Hb by agro‐ecological zone, ranging from 109.1 g/L (95% CI, [105.2, 113.0]) in the Coastal Savannah to 118.6 g/L (95% CI [117.1, 120.2]) in the forest zone (Table 2). Likewise, the mean Hb differed significantly between SAC and adolescents in each agro‐ecological zone except for the Northern Savannah zone, but the prevalence of anaemia did not differ significantly between SAC and adolescents in each agro‐ecological zone. The overall prevalence rate of anaemia varied from 36.4% in the forest zone to 63.8% in the Coastal Savannah with the odds of anaemia been significantly higher for all agro‐ecological zones compared with the forest zone (Table 2).

**Table 2:** Mean (95% CI) haemoglobin concentration (g/L) and prevalence of anaemia among the rural Ghanaian children stratified by age category and agro‐ecological zone

Zone	Mean (95% CI) of haemoglobin concentration (g/L)				Prevalence of anaemia			
	Overall sample	School age	Adolescents	*P* value	Overall sample	School age	Adolescents	*P* value
	*n* = 642	*n* = 323	*n* = 319		*n* = 642	*n* = 323	*n* = 319	
Forest zone (ref, *n* = 247)	118.6 (117.1, 120.2)	115.9 (113.7, 118.1)	121.2 (119.1, 123.4)	<0.001	36.4	39.7	33.3	0.301
Northern Savannah (*n* = 190)	110.5 (108.9, 112.2)^a^	109.8 (107.6, 111.9)^a^^,^ b	111.6 (109.1, 114.2)^a^	0.277	63.2^c^	62.2	64.6	0.736
Coastal Savannah (*n* = 58)	109.1 (105.2, 113.0)^a^	102.5 (96.9, 108.1)^a^	113.4 (108.5, 118.4)^a^	0.005	63.8^c^	69.6	60.0	0.458
Transitional zone (*n* = 147)	111.7 (109.7, 113.8)^a^	109.2 (106.1, 112.2)^a^	113.9 (111.2, 116.6)^a^	0.023	60.5^c^	66.2	55.7	0.195
Overall sample (*n* = 642)	113.8 (112.8, 114.8)	111.4 (110.0, 112.8)	116.2 (114.7, 117.6)	<0.001	52.3	55.1	49.5	0.16

*Note*. *n*: sample size; ref.: reference group; 95% CI: 95% confidence interval; one‐way analysis of variance was used for statistical difference in haemoglobin concentration between age groups whereas the chi square/Fisher's exact test was appropriately used for the prevalence of anaemia.

a Statistically significantly lower (*P* < 0.05) than the forest zone (Tukey–Kramer adjustment).

b Statistically significantly higher (*P* < 0.05) than the Coastal Savannah zone category (Tukey–Kramer adjustment).

c Odds of anaemia were significantly higher with reference to forest zone (*P* trend <0.001).

Additionally, the results of the bivariate linear regression (Table S1) showed that child's age, school grade, ethnicity (Gurma vs. Akan), the number of days child bought food from school, HDDS, HAFC, and agro‐ecological zone were significant correlates of the children's Hb status.

### 3.3 Results of the multiple regressions

In the backward multiple linear regression (Table 3), we identified child's age (β = 1.21, *P* < 0.001), HDDS (β = 0.59, *P* = 0.033), and agro‐ecological zone (*P* trend < 0.001) as significant predictors of the children's Hb. The effects of farm diversity and household asset index (HAI) were not statistically significant for the whole population. When stratifying by age category, the HAI (*P* trend = 0.042) and agro‐ecological zone (*P* trend < 0.001) were the only significant predictors of adolescents' Hb, whereas child's age (β = 2.21, *P* < 0.001), farm diversity (β = 0.59, *P* = 0.036), and agro‐ecological zone (*P* trend < 0.001) significantly predicted Hb in SAC.

**Table 3:** Backward multiple linear regression of the factors associated with haemoglobin concentration among rural Ghanaian school‐aged children and adolescents

Factors	Overall *n* = 642			School‐aged children (6–9 years) *n* = 323			Adolescents (10–17 years) *n* = 319		
	β	SE (β)	*P* value	β	SE (β)	*P* value	β	SE (β)	*P* value
Age^a^	1.21	0.23	<0.001	2.21	0.62	<0.001			
Dependency ratio^b^				−5.85	4.03	0.149			
HDDS^c^	0.59	0.28	0.033						
Farm diversity^a^	0.37	0.21	0.059	0.59	0.30	0.036			
Household asset index^d^			0.099						0.042
Lower	Ref.						Ref.		
Middle	1.84	1.20					4.22	1.74	
Upper	−0.80	1.29					0.80	1.87	
Ecological zone			<0.001			<0.001			<0.001
Forest	Ref.			Ref.			Ref.		
Northern Savannah	−8.00	1.28		−6.18	1.63		−9.71	1.94	
Coastal Savannah	−8.61	1.95		−12.22	2.95		−7.11	2.46	
Transition	−7.04	1.32		−5.50	1.92		−7.56	1.83	
Model fit statistics									
*F*	13.11		<0.001	9.08		<0.001	8.21		<0.001
*R* ^2^_adjusted_	0.14			0.14			0.11		
MSE	148.54			142.50			149.78		
AIC	3,647.43			1850.36			1792.66		
PRESS	91,386			45077			45496		

*Note*. *n*: sample size; β: regression coefficient; SE (β): standard error of regression coefficient; ref: reference group; MSE: mean square of residuals; AIC: Akaike criteria; PRESS: Predicted Residual Sum of Squares Statistic.

a Selected for full sample and school‐aged children (SAC).

b Selected for only SAC.

c Selecteed for only full sample.

d Selected for full sample and adolescents.

Compared with the forest zone, children residing in other agro‐ecological zones consistently had a higher odds of anaemia both at the population level and by age category (*P* trend < 0.001 for all models). Furthermore, HDDS (POR = 0.91, 95% CI [0.83, 1.00]) and availability of maize stock in the household (POR = 0.70, 95% CI [0.47, 1.04]) were associated with lower odds of anaemia for the full sample. Additionally, availability of maize stock in the household was significantly associated with a 45% lower odds of anaemia among SAC. Although a unit increase in HDDS was associated with a lower odds of anaemia among SAC, the effect was not statistically significant. Moreover, the middle tercile of the agriculture asset index was associated with 52% higher odds of anaemia whereas the upper tercile was associated with 26% lower odds of anaemia among SAC, but none was statistically significant. Although a unit increase in age was associated with a 23% (POR = 0.77, 95% CI [0.62, 0.96]) *lower* odds in anaemia among SAC, it was associated with a 24% *higher* odds in anaemia among adolescents (POR = 1.24, 95% CI [1.03, 1.49]; Table 4). There was a significant interaction term for age and sex indicating that the effect of age was only significant for female adolescents but not male adolescents (POR = 1.35, 95% CI [1.04, 1.76] vs. POR = 1.14, 95% CI [0.88, 1.46] for female and male adolescents, respectively).

**Table 4:** Backward multiple logistic regression of the factors associated with anaemia among rural Ghanaian school‐aged children and adolescents

Factors	Overall *n* = 642		School‐aged children (6–9 years) *n* = 323		Adolescents (10–17 years) *n* = 319	
	POR (95% CI)	*P* value	POR (95% CI)	*P* value	POR (95% CI)	*P* value
Age^a^			0.77 (0.62, 0.96)	0.022	1.24 (1.03, 1.49)	0.021
Sex^b^						0.273
Male					Ref.	
Female					0.32 (0.04, 2.47)	
Age * sex^c^						0.348
Age when sex = female					1.35 (1.04, 1.76)	
Age when sex = male					1.14 (0.88, 1.46)	
HDDS^d^	0.91 (0.83, 1.00)	0.054	0.89 (0.78, 1.02)	0.085		
Maize stock available in household^d^		0.074		0.038		
No	Ref.		Ref.			
Yes	0.70 (0.47, 1.04)		0.55 (0.32, 0.97)			
Agriculture asset index^e^				0.099		
Lower			Ref.			
Middle			1.52 (0.87, 2.65)			
Upper			0.74 (0.39, 1.41)			
Ecological zone		<0.001		<0.001		<0.001
Forest	Ref.		Ref.		Ref.	
Northern Savannah	3.23 (2.14, 4.89)		2.60 (1.48, 4.57)		3.68 (2.01, 6.71)	
Coastal Savannah	2.94 (1.58, 5.48)		3.54 (1.27, 9.86)		3.21 (1.46, 7.05)	
Transition	2.78 (1.80, 4.28)		3.10 (1.56, 5.81)		2.46 (1.36, 4.40)	
Model fit statistics						
Log likelihood ratio	50.13	<0.001	38.26	<0.001	32.58	<0.001
Wald test	46.90	<0.001	33.02	<0.001	29.36	<0.001
Nagelkerke's *R* ^2^	0.11		0.16		0.13	

*Note*. *n*: sample size; POR: prevalence odds ratio; 95% CI: 95% confidence interval; ref: reference group.

* in the “age * sex” in the factor column refers to the multiplication for the interaction term of age and sex.

a Not selected in full sample.

b Selected for only adolescent subsample.

c Interaction term selected for only adolescent subsample.

d Selected for full sample and school‐aged children (SAC).

e Selected for only SAC.

In Table 3, the proportion of variance explained (adjusted *R* ^*2*^) in the selected models for Hb was 14% for SAC and 13% for adolescents. Similarly, for anaemia (Table 4), the Nagelkerke's *R* ^*2*^ was 0.16 and 0.13 for SAC and adolescents, respectively.

## 4 DISCUSSION

To our knowledge, this is the first study to investigate the prevalence of anaemia as well as the predictors of Hb status and anaemia among rural SAC and adolescents in Ghana. About one in two children in our sample was found to be anaemic with the majority of children being moderately anaemic (36.0%), which signals the presence of a serious public health issue (WHO, 2011). Anaemia prevalence rates ranging between 21.1% and 82.6% have been reported for SAC and adolescents in Sub‐Saharan Africa (Assefa, Mossie, & Hamza, 2014; Bharati et al., 2009; Onimawa et al., 2010; Tesfaye et al., 2015). In countries like Ghana, poor dietary intake due to food insecurity and/or consumption of monotonous plant‐based diets and infections in rural settings are key drivers of inadequate micronutrient intake and anaemia (Righetti et al., 2012, 2013; Tatala, Ndossi, & Ash, 2004; Zimmermann, Chaouki, & Hurrell, 2005).

Compared with our results, a recent study in the transition zone of Ghana reported a lower prevalence rate (30.8%) of anaemia among rural 6‐ to 12‐year‐old SAC (Egbi et al., 2014). However, two recent studies in the Northern Savannah zone of Ghana reported a higher prevalence rate (64%) than our study among 5‐ to 12‐year‐old rural SAC (Abizari et al., 2017). Although the apparent differences with our findings may be related to differences in sample size and socio‐economic conditions among others, the results suggest a variation in the prevalence of anaemia based on geographic/contextual circumstances. Agro‐ecological zone consistently stood out as a key significant predictor of both Hb and anaemia in all models. Specifically, residing in any other agro‐ecological zone compared with the forest zone was associated with a significant reduction in Hb level and a higher odds of anaemia (*P* trend < 0.001). The within‐country variations in anaemia prevalence may partly be attributed to geographical disparities in dietary patterns, prevalence, and incidence of diseases as well as socio‐economic factors. In Ghana, people residing in the Northern Savannah zone are notably more food insecure (WFP, 2009), consume fewer fruits than those in the Coastal Savannah and forest zones (Amo‐Adjei & Kumi‐Kyereme, 2015), and have a higher prevalence of malaria, which may be all contributing to anaemia (Otupiri, Yar, & Hindin, 2012). The finding corroborates that of Hall et al. (2009) who found a significant difference in the mean Hb and prevalence of anaemia between children residing by Lake Victoria and those residing on the coast of Tanzania.

In the present study, older children had higher levels of Hb, which has already been reported in other contexts (Assefa et al., 2014; Mesfin et al., 2015; Ngesa & Mwambi, 2014). However, in our stratified analysis, the observed trend remained statistically significant only for SAC, for whom a year increase in age was associated with a 2.21‐g/L increase in Hb and a 23% significant reduction in the odds of anaemia, suggesting that young age was a risk factor for SAC. Younger children are generally more vulnerable to poor health as they are often more at risk of dietary inadequacy and infections such as malaria and helminths; for instance, poor dietary diversity and a high prevalence of malaria (81.3%) and subclinical inflammation (48.7%) have been reported among rural SAC in Ghana (Abizari et al., 2017).

Age was not significantly associated with the Hb status of adolescents in the multiple regression; however, we found that after peaking at 11 years, the Hb of females declined (Figure S1a), which corroborates the findings of Sabale, Kowli, and Chowdary (2013) who found that Hb decreases significantly as age increasing from 9–15 years among Mumbai school girls aged 9–19 years. The mean age of the adolescent girls (11.2 ± 1.3 years) in the present study corresponded with the mean age of the onset of menarche in Ghana (Gumanga & Kwame‐Aryee, 2012; Richmond, Anthony, & Martin, 2011) and may partly explain our finding. Unfortunately, we could not further test this hypothesis, as the baseline data did not include information on age at menarche.

Furthermore, a unit increase in age was significantly associated with 24% higher odds of anaemia for adolescents in the multiple logistic regression. However, the inclusion of an interaction term for age and sex showed that the odds of anaemia for a unit increase in age was only significantly higher for adolescent girls and not boys. This is not surprising considering the growth spurt in early adolescence and the increased iron requirements for girls at menarche (WHO/FAO, 2004). Indeed, adolescent girls are particularly at high risk of developing iron deficiency and/or anaemia, especially among those who experience heavy blood losses during menstruation and corresponding decreases in ferritin levels (Black et al., 2008; Chaparro & Lutter, 2008). The finding emphasizes the need for intervention programmes to curtail anaemia in early adolescence to compensate for the additional nutrient requirements for growth and puberty as well as the extra losses due to menstruation.

**Figure 1:**
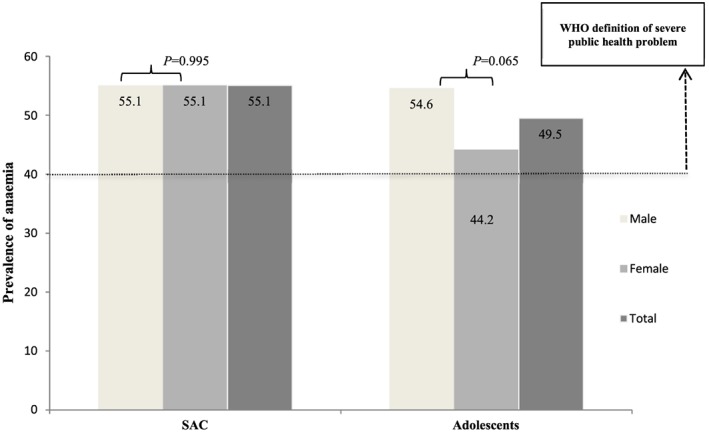
The prevalence of anaemia among the rural Ghanaian school children stratified by age category and sex. SAC: school‐aged children

In contrast to many other studies that have reported a higher prevalence and risk of anaemia among females compared with males (Egbi et al., 2014; Salama & Labib, 2016; Tesfaye et al., 2015), though not significant, our findings pointed to a higher prevalence of anaemia among adolescent males compared with females and a similar prevalence for both school‐age males and females (Figure 1). The finding is nevertheless similar to the result of Hall et al. (2009), who found a higher prevalence of anaemia for 12‐ to 14‐year‐old boys compared with girls in rural Volta region of Ghana. Although the mean Hb of adolescent males was slightly lower than that of adolescent females in the present study (boys: 115.3 g/L, girls: 117.0 g/L, *P* = 0.978), the WHO criteria classifies adolescent males aged ≥15 years as anaemic at a higher threshold compared with adolescent females with similar age (130 vs. 120 g/L). Thus, more adolescent males than females were classified as anaemic and may partly account for the finding. Nonetheless, the implication of this finding is that adolescent boys should also be targeted for anaemia control programmes besides adolescent girls, the usual priority group.

In agreement with evidence that high socio‐economic status is protective of anaemia (Abdel‐Rasoul, El Bahnasy, El Shazly, Gabr, & Abdel‐Aaty, 2015; Ngesa & Mwambi, 2014), belonging to the middle or upper terciles of the HAI was associated with a significant increase in Hb compared with those in the lower tercile of the HAI for adolescents. In addition, a unit increase in HDDS was associated with a 0.59‐g/L significant increase in the Hb level of the children, but the association did not hold in the stratified analysis by age category, which could be because the subsamples were underpowered to detect any statistical certainty. Farm diversity seemed to have a weak association with Hb, but the association was clearer for SAC for whom a unit increase in farm diversity was significantly associated with a 0.59‐g/L increase in Hb. We found no interaction between farm diversity and HDDS in the present study; nevertheless, studies have shown that farm diversity improves household dietary diversity (Jones et al., 2014; Sibhatu et al., 2015). The HDDS is a measure of household food security rather than dietary quality (Ruel, 2003), but it has been shown that household food security influences the quality of individual diets and micronutrient adequacy (Leung, Epel, Ritchie, Crawford, & Laraia, 2014; Tarasuk & Beaton, 1999). SAC children in households with maize stock were less likely to have anaemia compared with their peers in households without maize stock. Maize is generally a staple crop in most Ghanaian homes, and food secured households are most likely to have maize stock in the household year round; this suggests that SAC may be more vulnerable to the effects of household food insecurity, compared with adolescents.

Several studies have reported that parental education has a protective effect against anaemia in resource‐poor settings (Assefa et al., 2014; Ngesa & Mwambi, 2014; Tesfaye et al., 2015); therefore, it is surprising that this association does not hold in our study with education as either a categorical variable or a continuous variable. This may be due to the relative lack of variation in educational status in our sample, with most of the parents having completed only a few years of schooling (mothers: 3.1 ± 5.6 years, fathers: 5.6 ± 6.5 years). Likewise, we did not find any association between maternal occupation and paternal occupation with Hb status or anaemia in contrast to several other studies (Assefa et al., 2014; Mesfin et al., 2015; Tesfaye et al., 2015).

Even though helminths infestation is a key significant determinant of anaemia (Leenstra et al., 2004; Salama & Labib, 2016), remarkably, we did not find any helminths infestation among a random subsample of the children (*n* = 311) that was partly attributable to the mass deworming of school children, which is being implemented in Ghana by the Ghana Health Service since 2009. Ghana started its national deworming campaign for schistosomiasis and soil‐transmitted helminths in that year. Indeed, schools can constitute effective platforms for reaching this population through integrated deworming, water, hygiene, and sanitation programmes (Bundy, 2011).

### 4.1 Strengths and limitations of the study

The results were generally robust when modelling with backward regression, multilevel regression, and Ordinary Least Squares (OLS). Nonetheless, some limitations inherent in the present study should be considered in the interpretation of our findings. Firstly, because these are observational, cross‐sectional data, these cannot be interpreted as causal findings. We, therefore, limit our interpretation to describing associations. Even so, we thoroughly modelled several potential explanatory variables including subject and household demographics, education, livelihood, wealth, farming and production orientation, and quality of household dietary intake, as well as used different methods to test the robustness of these associations.

Furthermore, intakes of vitamin A‐rich and iron‐rich foods are well‐recognized predictors of anaemia (Zimmermann et al., 2005), yet it was not possible to evaluate the contribution of dietary intake to the improvement of Hb in the present study as we lacked data on intakes of these foods specifically. Malaria is another factor that may contribute to anaemia (Leenstra et al., 2004; WHO, 2001), and a high prevalence of malaria among Ghanaian children has previously been reported (Abizari et al., 2017; Otupiri et al., 2012). Although we did not include malaria incidence in our statistical models, child sickness in the last 7 days preceding the survey was captured, which we presume, may have sufficiently accounted for any malaria incidence.

The selected final models accounted for smaller proportions of the variance for Hb and anaemia; nevertheless, this does not necessarily weaken the effect and the importance of the significant predictors to our outcomes. The implication of the relatively small percentage of variance explained by our models may be that, there are other unaccounted predictors contributing to the unexplained variances, which we have tried discussing. Overall, the *F* value (for linear regression) and log‐likelihood ratio (logistic regression) for all the models were statistically significant with *P* values <0.001.

Our study population is rural, which limits the generalization of our findings to all SAC and adolescents in Ghana. Moreover, only SAC and adolescents enrolled in school were studied, but studies have shown that nonenrolled children may be more anaemic than those in school because enrolled children are in a better position to understand health and nutritional risks as this could be taught in school or sensitization programmes may be carried out in their schools. This could have underestimated the true prevalence of anaemia in our study, but considering that 36% of children in rural areas never enter school in Ghana (UNICEF‐Ghana, 2015), the study population may well represent all rural children.

## CONFLICTS OF INTEREST

The authors declare that they have no conflicts of interest.

## CONTRIBUTIONS

AG, GF, and EA conceived and designed the study. AG, GF, KMB, and IA contributed to the survey tools. GF, KMB, and IA performed the data collection. FA conducted the statistical analyses. FA and EA wrote the first draft of the manuscript. AG, IB, SO, GF, KMB, and IA contributed to the writing of the manuscript. EA and IB are primarily responsible for the final content. All authors read and approved the final manuscript.

## Supporting information

Table S1: Bivariate regression of the factors associated with haemoglobin concentration among rural Ghanaian schoolchildren stratified by age category
Table S2: Univariate logistic regression of the factors associated with anaemia among rural Ghanaian schoolchildren stratified by age category
*Figure S1: A smoothed scatter plot of the haemoglobin concentration(hb_gdl) of the school age children and adolescents by sex*; *females (a) and males (b); interpret with caution as sample size from 14 to 17 years were small (14 y, n = 11; 15 y, n = 2; 16 y, n = 2 and 17 y, n = 1)*
*Figure S2: Prevalence of anaemia among school‐age children by agro‐ecological zone and sex*
*Figure S3: Prevalence of anaemia among adolescents by agro‐ecological zone and sex*
