# Supplementary material for: Agro‐ecological zone and farm diversity are factors associated with haemoglobin and anaemia among rural school‐aged children and adolescents in Ghana
Source: Matern Child Nutr. 2018 Jul 25;15(1):e12643. doi: 10.1111/mcn.12643 (PMC7198936; doi:10.1111/mcn.12643)
Supplement: Supplementary file 1 — Table S1: Bivariate regression of the factors associated with haemoglobin concentration among rural Ghanaian schoolchildren stratified by age category Table S2: Univariate logistic regression of the factors associated with anaemia among rural Ghanaian schoolchildren stratified by age category Figure S1: A smoothed scatter plot of the haemoglobin concentration(hb_gdl) of the school age children and adolescents by sex; females (a) and males (b); interpret with caution as sample size from 14 to 17 years were small (14 y, n = 11; 15 y, n = 2; 16 y, n = 2 and 17 y, n = 1) Figure S2: Prevalence of anaemia among school‐age children by agro‐ecological zone and sex Figure S3: Prevalence of anaemia among adolescents by agro‐ecological zone and sex [file MCN-15-e12643-s001.docx]

**Supplementary Material**

Table S1: Bivariate regression of the factors associated with haemoglobin concentration among rural Ghanaian schoolchildren stratified by age category

| **Predictors** | **Overall**  **n=642** | | | **School-age children (6-9 y)**  **n=323** | | | **Adolescents (10-17 y)**  **n=319** | | |
| --- | --- | --- | --- | --- | --- | --- | --- | --- | --- |
|  | **β** | **SE (β)** | ***P-value*** | **β** | **SE (β)** | ***P-value*** | **β** | **SE (β)** | ***P-value*** |
| **Child characteristics** |  |  |  |  |  |  |  |  |  |
| Sex |  |  |  |  |  |  |  |  |  |
| Female | Ref. | - |  | Ref. | - | - | Ref. | - | - |
| Male | 0.75 | 1.03 | 0.468 | -0.23 | 1.42 | 0.871 | 1.68 | 1.45 | 0.247 |
| Age | 1.25 | 0.23 | <0.001 | 2.22 | 0.63 | 0.001 | 0.30 | 0.55 | 0.593 |
| Child sick in the past 7 days |  |  |  |  |  |  |  |  |  |
| No | Ref. | - | - | Ref. | - | - | Ref. | - | - |
| Yes | -0.50 | 1.32 | 0.703 | -0.76 | 1.79 | 0.670 | 0.16 | 1.91 | 0.934 |
| Child’s birth order (continuous) | 0.10 | 0.24 | 0.678 | 0.10 | 0.35 | 0.648 | 0.15 | 0.32 | 0.772 |
| Child’s school grade |  |  |  |  |  |  |  |  |  |
| Lower Primary | Ref. | - | - | Ref. | - | - | Ref. | - | - |
| Upper primary and JHS | 4.35 | 1.38 | 0.002 | 8.75 | 4.86 | 0.073 | 1.55 | 1.57 | 0.325 |
| Ethnicity |  |  |  |  |  |  |  |  |  |
| Akan | Ref. | - | - | Ref. | - | - | Ref. | - | - |
| Gurma | -2.60 | 1.30 | 0.046^-^ | -2.44 | 1.77 | 0.170 | -2.07 | 1.87 | 0.269 |
| Mole-Dagbani | -2.58 | 1.48 | 0.081 | -2.42 | 2.08 | 0.245 | -2.69 | 2.04 | 0.188 |
| Other^a^ | -2.82 | 1.48 | 0.057 | -1.71 | 2.06 | 0.408 | -3.75 | 2.07 | 0.070 |
| Number of days child bought food from school | 0.47 | 0.24 | 0.052^-^ | 0.34 | 0.32 | 0.288 | 0.45 | 0.35 | 0.192 |
| **Household demographic and socioeconomic** **characteristics** |  |  |  |  |  |  |  |  |  |
| Dependency ratio | -2.74 | 2.78 | 0.324 | -6.05 | 4.03 | 0.135 | -0.16 | 3.73 | 0.967 |
| Sex of household head |  |  |  |  |  |  |  |  |  |
| Male | Ref. | - | - | Ref. | - | - | Ref. | - | - |
| Female | -0.05 | 1.15 | 0.966 | -1.29 | 1.58 | 0.417 | 1.15 | 1.61 | 0.475 |
| Age of household head | 0.06 | 0.04 | 0.161 | 0.11 | 0.06 | 0.042 | -0.01 | 0.05 | 0.917 |

***Table S1: Cont.***

| **Predictors** | **Overall**  **n=642** | | | **School-age children (6-9 y)**  **n=323** | | | **Adolescents (10-17 y)**  **n=319** | | |
| --- | --- | --- | --- | --- | --- | --- | --- | --- | --- |
|  | **β** | **SE (β)** | ***P-value*** | **β** | **SE (β)** | ***P-value*** | **β** | **SE (β)** | ***P-value*** |
| Household asset index |  |  |  |  |  |  |  |  |  |
| Lower | Ref. | - |  | Ref. | - | - | Ref. | - | - |
| Middle | 1.89 | 1.26 | 0.133 | -0.11 | 1.73 | 0.950 | 3.72 | 1.78 | 0.160 |
| Upper | 0.17 | 1.26 | 0.891 | -2.38 | 1.73 | 0.169 | 2.50 | 1.78 | 0.037 |
| School years of father | 0.11 | 0.08 | 0.175 | 0.16 | 0.11 | 0.154 | 0.04 | 0.11 | 0.701 |
| School years of mother | -0.07 | 0.09 | 0.430 | -0.24 | 0.13 | 0.073 | 0.02 | 0.13 | 0.881 |
| Occupation of father (n=429) |  |  |  |  |  |  |  |  |  |
| Famer | Ref | - | - | Ref | - | - | Ref | - | - |
| Other^b^ | 1.74 | 1.35 | 0.199 | -1.02 | 2.05 | 0.618 | 2.46 | 1.81 | 0.176 |
| Occupation of mother (n=596) |  |  |  |  |  |  |  |  |  |
| Farmer | Ref. | - | - | Ref. | - | - | Ref. | - | - |
| Trader | 1.14 | 1.14 | 0.316 | 1.39 | 1.55 | 0.371 | 0.42 | 1.62 | 0.797 |
| Other^c^ | -0.41 | 1.46 | 0.782 | -0.13 | 1.97 | 0.947 | -0.93 | 2.10 | 0.657 |
| Household received some remittance in the past 1 year |  |  |  |  |  |  |  |  |  |
| No | Ref. | - | - | Ref. | - | - | Ref. | - | - |
| Yes | -0.31 | 1.23 | 0.789 | -1.44 | 1.58 | 0.364 | 0.34 | 1.56 | 0.827 |
| **Household food availability and diet diversity** |  |  |  |  |  |  |  |  |  |
| HDDS | 0.55 | 0.27 | 0.044 | 0.54 | 0.38 | 0.157 | 0.50 | 0.38 | 0.183 |
| HFVS | 0.14 | 0.08 | 0.110 | 0.10 | 0.12 | 0.427 | 0.14 | 0.11 | 0.224 |
| HAFC | 0.89 | 0.43 | 0.036 | 1.03 | 0.66 | 0.119 | 0.77 | 0.68 | 0.254 |
| Proportion of food consumed in the past month from own production^d^ | 0.19 | 0.31 | 0.541 | 0.88 | 0.45 | 0.052 | -0.02 | 0.41 | 0.967 |
| Number of months household consumed food from own production | 0.04 | 0.12 | 0.732 | 0.19 | 0.18 | 0.310 | 0.08 | 0.16 | 0.604 |
| Maize stock in household |  |  |  |  |  |  |  |  |  |
| No | Ref. | - | - | Ref. | - | - | Ref. | - | - |
| Yes | 0.89 | 1.22 | 0.467 | 3.16 | 1.64 | 0.055 | -1.24 | 1.74 | 0.479 |
| Farm diversity | 0.33 | 0.20 | 0.103 | 0.86 | 0.27 | 0.002 | -0.03 | 0.28 | 0.913 |
| Household agriculture asset index |  |  |  |  |  |  |  |  |  |
| Lower | Ref. | - | - | Ref. | - | - | Ref. | - | - |
| Middle | 0.66 | 1.15 | 0.568 | -0.82 | 1.60 | 0.611 | 2.28 | 1.61 | 0.157 |
| Upper | 1.62 | 1.42 | 0.257 | 2.20 | 1.91 | 0.250 | 1.47 | 2.07 | 0.479 |

***Table S1: Cont.***

| **Predictors** | **Overall**  **n=642** | | | **School-aged children (6-9 y)**  **n=323** | | | **Adolescents (10-17 y)**  **n=319** | | |
| --- | --- | --- | --- | --- | --- | --- | --- | --- | --- |
|  | **β** | **SE (β)** | ***P-value*** | **β** | **SE (β)** | ***P-value*** | **β** | **SE (β)** | ***P-value*** |
| Household owns land |  |  |  |  |  |  |  |  |  |
| No | Ref. | - | - | Ref. | - | - | Ref. | - | - |
| Yes | 0.29 | 1.25 | 0.814 | 1.69 | 1.84 | 0.360 | 0.03 | 1.67 | 0.986 |
| Geographical location |  |  |  |  |  |  |  |  |  |
| Ecological zone in Ghana |  |  |  |  |  |  |  |  |  |
| Forest | Ref. | - | - |  | Ref. | - | Ref. | - | - |
| Northern Savannah | -8.08 | 1.21 | <0.001 | -6.17 | 1.60 | <0.001 | -9.57 | 1.77 | <0.001 |
| Coastal Savannah | -9.54 | 1.83 | <0.001 | -13.46 | 2.77 | <0.001 | -7.78 | 2.35 | 0.001 |
| Transitional | -6.91 | 1.30 | <0.001 | -6.77 | 1.85 | <0.001 | -7.31 | 1.77 | <0.001 |

β,regression coefficient ; SE (β), standard error of regression coefficient; ref, reference group; HDDS, household dietary diversity score; HFVS, household food variety score; HAFC, household animal foods consumption; ^a^Other includes Ga-Dangbe, Guan, Grusi, Mande, Ewe and other tribes originating from outside Ghana; ^b^Other includes off-farm wage employment, business and unemployed; ^c^Other includes off-farm wage employment, apprentice and unemployed; ^d^Natural log-transformed variable of proportion of food consumed in the past month from own production;

Table S2: Univariate logistic regression of the factors associated with anaemia among rural Ghanaian schoolchildren stratified by age category

| **Predictors** | **Overall**  **n=642** | | **School-age children (6-9 y)**  **n=323** | | **Adolescents (10-17 y)**  **n=319** | |
| --- | --- | --- | --- | --- | --- | --- |
|  | **POR (95% C.I)** | ***P*-value** | **POR (95% C.I)** | ***P*-value** | **POR (95% C.I)** | ***P*-value** |
| **Child characteristics** |  |  |  |  |  |  |
| Sex |  |  |  |  |  |  |
| Female | Ref. |  | Ref. |  | Ref. |  |
| Male | 1.23 (0.90, 1.68) | 0.190 | 1.00 (0.64, 1.55) | 0.995 | 1.52 (0.98, 2.36) | 0.065 |
| Age | 0.96 (0.89, 1.03) | 0.271 | 0.77 (0.63, 0.95) | 0.012 | 1.22 (1.03, 1.45) | 0.023 |
| Child sick in the past 7 days |  |  |  |  |  |  |
| No | Ref. |  | Ref. |  | Ref. |  |
| Yes | 1.01 (0.68, 1.51) | 0.954 | 1.16 (0.66, 2.01) | 0.610 | 0.86 (0.48, 1.53) | 0.610 |
| Child’s birth order (continuous) | 0.98 (0.92, 1.06) | 0.652 | 1.00 (0.91, 1.11) | 0.984 | 0.96 (0.87, 1.07) | 0.472 |
| Child’s school grade |  |  |  |  |  |  |
| Lower | Ref. |  | Ref. |  | Ref. |  |
| Upper primary and JHS | 0.84 (0.55, 1.27) | 0.398 | 0.32 (0.06, 1.67) | 0.175 | 1.03 (0.64, 1.65) | 0.911 |
| Ethnicity |  | 0.021 |  | 0.072 |  | 0.125 |
| Akan | Ref. |  | Ref. |  | Ref. |  |
| Gurma | 1.72 (1.16, 2.55) |  | 2.04 (1.17, 3.56) |  | 1.39 (0.78, 2.45) |  |
| Mole,Dagbani | 1.38 (0.87, 2.16) |  | 1.74 (0.91, 3.33) |  | 1.12 (0.60, 2.08) |  |
| Other^a^ | 1.75 (1.12, 2.75) |  | 1.44 (0.76, 2.72) |  | 2.13 (1.12, 4.06) |  |
| Number of days child bought food from school | 0.94 (0.87, 1.01) | 0.074 | 0.93 (0.84, 1.03) | 0.163 | 0.95 (0.85, 1.05) | 0.319 |
| **Household demographic and socioeconomic characteristics** |  |  |  |  |  |  |
| Dependency ratio | 1.54 (0.66, 3.55) | 0.316 | 2.65 (0.75, 9.34) | 0.132 | 1.00 (0.32, 3.09) | 0.998 |
| Sex of household head |  |  |  |  |  |  |
| Male | Ref. |  | Ref. |  | Ref. |  |
| Female | 0.91 (0.64, 1.28) | 0.573 | 0.96 (0.59, 1.57) | 0.881 | 0.85 (0.52, 1.39) | 0.522 |
| Age of household head | 0.99 (0.98, 1.00) | 0.189 | 0.98 (0.97, 1.00) | 0.062 | 1.00 (0.98, 1.02) | 0.992 |
| Household asset index |  | 0.655 |  | 0.791 |  | 0.159 |
| Lower | Ref. |  | Ref. |  | Ref. |  |
| Middle | 0.88 (0.61, 1.29) |  | 1.18 (0.69, 2.01) |  | 0.66 (0.38, 1.13) |  |
| Upper | 0.84 (0.58, 1.23) |  | 1.16 (0.68, 1.98) |  | 0.61 (0.36, 1.05) |  |
| School years of father | 0.98 (0.96, 1.01) | 0.157 | 0.98 (0.95, 1.02) | 0.339 | 0.98 (0.95, 1.02) | 0.315 |
| School years of mother | 1.00 (0.97, 1.03) | 0.996 | 1.01 (0.97, 1.05) | 0.756 | 1.00 (0.96, 1.04) | 0.873 |

***Table S2: Cont.***

| **Predictors** | **Overall**  **n=642** | | **School-age children (6-9 y)**  **n=323** | | **Adolescents (10-17 y)**  **n=319** | |
| --- | --- | --- | --- | --- | --- | --- |
|  | **POR (95% C.I)** | ***P*-value** | **POR (95% C.I)** | ***P*-value** | **POR (95% C.I)** | ***P*-value** |
| Occupation of father ( n=429) |  |  |  |  |  |  |
| Farmer | Ref. |  | Ref. |  | Ref. |  |
| Other^b^ | 0.90 (0.60, 1.35) | 0.605 | 1.29 (0.68, 2.43) | 0.439 | 0.73 (0.42, 1.28) | 0.267 |
| Occupation of mother (n=596) |  | 0.589 |  | 0.622 |  | 0.921 |
| Farmer | Ref. |  | Ref. |  | Ref. |  |
| Trader | 0.83 (0.58, 1.18) |  | 0.78 (0.47, 1.30) |  | 0.99 (0.52, 1.88) |  |
| Other^c^ | 0.92 (0.58, 1.44) |  | 0.86 (0.45, 1.64) |  | 0.91 (0.55, 1.49) |  |
| Household received some remittance in the past 1 year |  | 0.608 |  | 0.881 |  | 0.626 |
| No | Ref. |  | Ref. |  | Ref. |  |
| Yes | 0.92 (0.65, 1.28) |  | 0.96 (0.59, 1.57) |  | 0.89 (0.56, 1.42) |  |
| **Household food availability and diet diversity** |  |  |  |  |  |  |
| HDDS | 0.92 (0.85, 1.00) | 0.055 | 0.89 (0.78, 0.99) | 0.047 | 0.96 (0.86, 1.08) | 0.485 |
| HFVS | 0.98 (0.96, 1.01) | 0.187 | 0.97 (0.94, 1.01) | 0.178 | 0.99 (0.96, 1.03) | 0.635 |
| HAFC | 0.91 (0.79, 1.05) | 0.205 | 0.91 (0.74, 1.11) | 0.351 | 0.92 (0.75, 1.13) | 0.442 |
| Proportion of food consumed in the past month from own production^d^ | 1.02 (0.93, 1.12) | 0.717 | 0.96 (0.83, 1.10) | 0.553 | 1.05 (0.93, 1.19) | 0.443 |
| Number of months household consumed food from own production | 1.00 (0.96, 1.04) | 0.920 | 0.99 (0.94, 1.05) | 0.800 | 1.00 (0.95, 1.05) | 0.848 |
| Maize stock available in household |  |  |  |  |  |  |
| No | Ref. |  | Ref. |  | Ref. |  |
| Yes | 0.79 (0.55, 1.13) | 0.197 | 0.59 (0.35, 0.98) | 0.040 | 1.06 (0.63, 1.80) | 0.822 |
| **Agriculture and farm diversity** |  |  |  |  |  |  |
| Farm diversity | 0.98 (0.93, 1.04) | 0.581 | 0.90 (0.82, 0.99) | 0.024 | 1.06 (0.97, 1.15) | 0.197 |
| Household agriculture asset index |  | 0.139 |  | 0.126 |  | 0.318 |
| Lower | Ref. |  | Ref. |  | Ref. |  |
| Middle | 1.01 (0.71, 1.43) |  | 1.31 (0.79, 2.16) |  | 0.78 (0.48, 1.27) |  |
| Upper | 0.67 (0.43, 1.02) |  | 0.69 (0.38, 1.24) |  | 0.64 (0.34, 1.20) |  |
| Household owns land |  |  |  |  |  |  |
| No | Ref. |  | Ref. |  | Ref. |  |
| Yes | 1.14 (0.79, 1.67) | 0.484 | 1.04 (0.59, 1.83) | 0.900 | 1.19 (0.72, 1.98) | 0.499 |

***Table S2: Cont.***

| **Predictors** | **Overall**  **n=642** | | **School-age children (6-9 y)**  **n=323** | | **Adolescents (10-17 y)**  **n=319** | |
| --- | --- | --- | --- | --- | --- | --- |
|  | **POR (95% C.I)** | ***P*-value** | **POR (95% C.I)** | ***P*-value** | **POR (95% C.I)** | ***P*-value** |
| **Geographical location** |  |  |  |  |  |  |
| Ecological zone in Ghana |  | <0.001 |  | <0.001 |  | <0.001 |
| Forest | Ref. |  | Ref. |  | Ref. |  |
| Northern Savannah | 2.99 (2.02, 4.43) |  | 2.50 (1.47, 4.24) |  | 3.64 (2.02, 6.58) |  |
| Coastal Savannah | 3.07 (1.70, 5.57) |  | 3.48 (1.33, 9.08) |  | 3.00 (1.39, 6.49) |  |
| Transitional | 2.68 (1.76, 4.08) |  | 2.98 (1.60, 5.53) |  | 2.51 (1.41, 4.48) |  |

n=sample size; POR, prevalence odds ratio ;95% C.I, 95% confidence interval ; ref, reference group; HDDS, household dietary diversity score; HFVS, household food variety score; HAFC, household animal foods consumption; ^a^Other includes Ga-Dangbe, Guan, Grusi, Mande, Ewe and other tribes originating from outside Ghana; ^b^Other includes off-farm wage employment, business and unemployed; ^c^Other includes off-farm wage employment, apprentice and unemployed; ^d^Natural log-transformed variable of proportion of food consumed in the past month from own production.


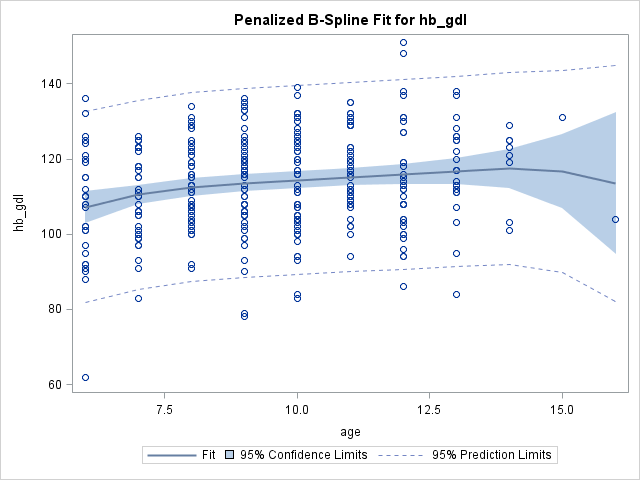


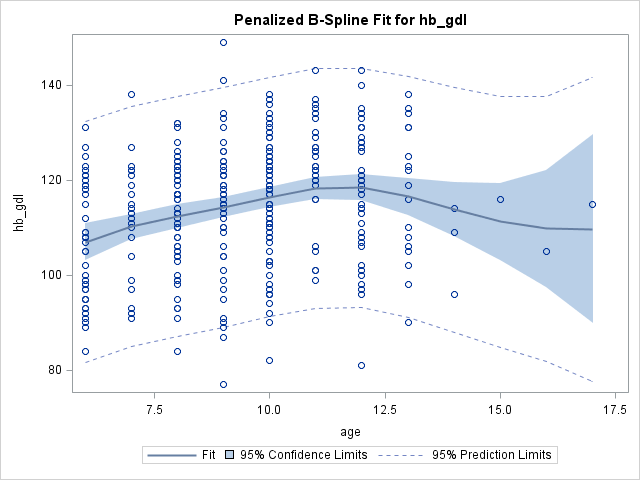


***Figure S1:***

b

a

***Figure S1:* *A smoothed scatter plot of the haemoglobin concentration( hb_gdl) of the school age children and adolescents by sex*;** *females (a) and males (b);* *interpret with caution as sample size from 14-17 years were small (14 y, n=11; 15 y, n=2; 16 y, n=2 and 17 y, n=1)*

**Severe public health problem**

*P*= 0.343

*P*= 0.551

*P*= 0.365

***Figure S2: Prevalence of anaemia among school-age children by agro-ecological zone and sex***

*P*=0.219

*P*=0.199

**Severe public health problem**

*P*=0.017

*P*=0.935

***Figure S3: Prevalence of anaemia among adolescents by agro-ecological zone and sex***
